# Whole-brain efferent projections of glutamatergic neurons in the cingulate cortex of mice

**DOI:** 10.1186/s13041-026-01290-6

**Published:** 2026-03-16

**Authors:** Fu Shi, Luyao Lei, Jiapeng Qiu, Ying Zhu, Haixing Wu, XiaoDan Wu

**Affiliations:** 1 Department of Anesthesiology, Shengli Clinical Medical College of Fujian Medical University, Fujian Provincial Hospital, Fuzhou University Affiliated Provincial Hospital, Fuzhou, Fujian China; 2 Fujian Provincial Key Laboratory of Critical Care Medicine, Fujian Provincial Hospital, Fuzhou University Affiliated Provincial Hospital, Fuzhou, Fujian China; 3 Department of Anesthesiology, Liaocheng People’s Hospital, Liaocheng City, Shandong Province China

**Keywords:** Cingulate cortex, Glutamatergic neuron, Efferent projection, Whole-brain mapping, Mice

## Abstract

The cingulate cortex, on the medial cerebral hemisphere, is involved in cognitive processing, emotional regulation, nociception, voluntary motor control, and sleep modulation. Anatomically, the cingulate cortex is organized into three distinct subdivisions: anterior cingulate cortex (ACC, A24a/A24b), midcingulate cortex (MCC, A24a'/A24b'), and posterior cingulate cortex (PCC, A30/A29c). Although emerging evidence from rodent models has mapped cingulate cortical projections, a comprehensive characterization of whole-brain efferent pathways from glutamatergic neurons in adult mice remains incomplete. In the present investigation, we applied a homologous nomenclature system and utilized viral anterograde tracing techniques, integrating both coronal and sagittal fluorescence imaging modalities, to systematically map and reconstruct the comprehensive efferent projections of glutamatergic neurons in the ACC, MCC, and PCC of adult mice. The findings revealed that the ACC, MCC, and PCC share a conserved projection architecture with direct efferent connections to key brain regions such as the intra-cingulate cortex, cerebral cortex, subcortical telencephalon, thalamus, and brainstem. Furthermore, our analysis revealed significant heterogeneity in the spatial distribution of efferent projections among cingulate subregions. Each subregion of the cingulate cortex exhibited distinct neuroanatomical connectivity patterns, which were posited to mediate their specialized functional roles. Neuroanatomical findings provided a fundamental basis for subsequent investigations into the functional roles of glutamatergic neurons in the cingulate cortex. Moreover, these findings provided a detailed structural framework that facilitated the elucidation of neural mechanisms underlying specific physiological processes.

## Introduction

The classification of cingulate area 1 (Cg1) and cingulate area 2 (Cg2), commonly employed in rodent models such as rats and mice, lacks phylogenetic homology with the anterior cingulate cortex (ACC) and midcingulate cortex (MCC) as defined in humans [1, 2]. This discrepancy is further compounded by the neuroanatomical interpretation in rodents, where the MCC has historically been regarded as a caudal extension of the ACC, thereby precluding its investigation as an independent structural entity. The application of ACC/MCC definitions facilitates the standardization of research findings across species in cingulate cortex studies, while simultaneously elucidating distinct functional and neural connectivity patterns among subregions within the rodent cingulate cortex. In this study, we adopted a homologous nomenclature system to analyze the whole-brain projections of the ACC (A24a/A24b), MCC (A24a'/A24b'), and PCC (A30/A29c). The implementation of this homologous nomenclature framework is anticipated to enhance the precision of functional characterization and enable more robust comparative analyses across the mammalian cingulate cortex.

Emerging evidence shows the cingulate cortex, involved in pain processing, emotional regulation, attention, cognition, and sensory integration, is critically linked to pathogenesis of various neurological and psychiatric disorders. Therapeutic strategies, including deep brain stimulation targeting the dorsal anterior cingulate cortex (dACC), have demonstrated significant efficacy in mitigating chronic refractory neuropathic pain [3]. Recent findings from the Lee group have elucidated that the neural circuit linking the ACC to the dorsolateral and lateral periaqueductal gray (dl/lPAG) modulates the amplification of pain-associated excessive fear [4]. Functional MRI studies, combined with orbicularis electromyography (EMG) assessments, have revealed that conditioned threat stimuli elicit rapid increases in ACC activity [5]. Notably, the MCC has been identified as a distinct subregion of the ACC in rodent models, characterized by unique cytoarchitecture, neurochemical profiles, and connectivity patterns. This region is implicated in diverse functions, including pain modulation, attentional control, decision-making, posttraumatic stress disorder, and sensory processing [6]. Specifically, the anteromedial thalamus-MCC CaMKIIα pathway has been shown to mediate pain and anxiety-like behaviors [7]. Furthermore, accumulating evidence highlights the role of the PCC in regulating attention, conscious awareness, and cognitive processes through its interactions with other brain networks [8]. Clinical studies have identified hypoconnectivity between the PCC and the ventromedial prefrontal cortex as a potential mechanism underlying impaired social signal interpretation and cognitive inflexibility in autism spectrum disorder [9].

The cingulate cortex is predominantly composed of pyramidal neurons, while its inhibitory interneurons are primarily parvalbumin (PV)- and somatostatin (SST)- expressing subtypes [10]. Pyramidal neurons in cingulate cortex are the primary excitatory neurons and utilize glutamate as their neurotransmitter. In the cingulate cortex, glutamatergic neurons are responsible for information processing, forming the excitatory backbone that integrates and propagates signals within local and long-range networks [11]. To comprehensively understand the diverse functions of the cingulate cortex in both physiological and pathological states, it is essential to map its anatomical projections in experimental animal models. Neural pathway tracing techniques, employing agents such as fluorogold (FG) or biotinylated dextran amine (BDA), have been utilized to delineate the neuronal pathways of the cingulate cortex through retrograde or anterograde transport mechanisms. Fillinger et al. [12, 13] identified a consistent projection pattern from the ACC and MCC, albeit with varying densities. In mice, neurons in the ACC and MCC showed extensive connections with cortical regions, basal forebrain, thalamus, hypothalamus, and brainstem. In rats, PCC neurons projected to the dorsal striatum, ventrolateral geniculate nucleus, intermedial zone, prefrontal nucleus, superior colliculus, periaqueductal gray matter, and brainstem [14]. However, due to neuronal heterogeneity in the cingulate cortex and the non-specific labeling of FG and BDA, these studies could not identify or precisely characterize efferent projection patterns of distinct neuronal subpopulations, particularly glutamatergic neurons.

A prior investigation employed adeno-associated virus (AAV) to systematically delineate ACC neurons receiving direct afferent inputs from distinct thalamic subnuclei in adult mice [15]. Our study utilized AAV vectors encoding CaMKIIα, a specific molecular marker for excitatory pyramidal neurons, to achieve selective targeting of cortical glutamergic neurons. AAV-CaMKIIα-mCherry-WPREs was used to map whole-brain efferent projections of cingulate cortex glutamatergic neurons in adult male mice through systematic fluorescence imaging of serially sectioned brain tissue after precise viral injections into the ACC, MCC, and PCC.

## Materials and methods

### Animals

Adult male C57BL/6 J mice, aged 6–8 weeks, were obtained from SPF (Suzhou) Biotechnology Co., Ltd. The animals were randomly allocated and maintained in standard plastic cages under controlled environmental conditions, including a 12-h light/dark cycle, ambient temperature of 22–25 °C, and ad libitum access to food and water. Following one-week acclimatization period, stereotaxic microinjections of adeno-associated virus (AAV) were precisely administered into the targeted brain regions. All experimental procedures involving animals were conducted in strict accordance with the ethical guidelines and protocols approved by the Institutional Animal Care and Use Committee of Fujian Medical University (the approval number IACUC FJMU 2025-0120).

### Virus anterograde tracing

Our study used 12 adult male C57BL/6 J mice. We assigned 4 mice to each cingulate cortex subregion (ACC, MCC, PCC). To examine the efferent projections of glutamatergic neurons in the cingulate cortex, we conducted stereotaxic microinjections of 200 nL of rAAV-CaMKIIα-mCherry-WPREs-pA viral vector (AAV2/9 serotype, 2.0 × 10^12^ genome copies/mL; Wuhan Brain Science Company) into the bilateral ACC, MCC, and PCC regions, respectively.

Briefly, twelve mice were anesthetized through intraperitoneal administration of 10% chloral hydrate (400 mg/kg; Leagene Biotechnology Co., Ltd., China) and subsequently immobilized in a stereotaxic frame (RWD Life Science Co., Ltd., China). Following subcutaneous administration of 0.2 ml of 2% lidocaine for local analgesia, the cranial surface was exposed, and a burr hole was created at the stereotaxic coordinates corresponding to the target brain region. Viral delivery was performed using a microinfusion pump (World Precision Instruments, USA) at a constant rate of 50 nl/min. After injection, the glass capillary was maintained in situ for 10 min to ensure optimal viral particle diffusion and to minimize potential reflux along the injection tract. Stereotaxic coordinates for the ACC (0.8 mm anterior to bregma, 0.2 mm lateral to midline, 1.6 mm ventral from the brain surface), MCC (0.47 mm posterior to bregma, 0.2 mm lateral to midline, 1.3 mm ventral from the brain surface), and PCC (2 mm posterior to bregma, 0.2 mm lateral to midline, 1.2 mm ventral from the brain surface) were determined according to the Mouse Brain Atlas, Fourth Edition.

Following the injection procedure, the surgical incision was carefully closed using sterile sutures and subsequently disinfected to minimize the risk of infection. Animals were returned to their housing unit after regaining full consciousness. Postoperative monitoring was conducted over a five-week period to verify adequate viral expression in both neuronal cell bodies and axonal projections.

### Slice preparation

Five weeks post-AAV injection, mice were subjected to transcardial perfusion with 0.01 M phosphate-buffered saline (PBS), followed by 4% paraformaldehyde (PFA) for tissue fixation. Subsequently, brain samples were excised and immersed in 4% PFA at 4 °C for overnight fixation, followed by dehydration in a 30% sucrose solution for 48 h. A total of 12 brain tissues were collected. In each cingulate cortex subregion (n = 4), two brains underwent coronal sectioning with one in anterior-to-posterior direction and the other in posterior-to-anterior direction; additionally, two brains were subjected to sagittal sectioning, one from midline to lateral aspect and the other from lateral aspect to midline. Coronal sections (ranging from + 4.0 mm anterior to − 8.0 mm posterior relative to bregma) or sagittal sections (ranging from 0 to 4 mm relative to the midline) were obtained at 30 µm thickness using a Leica CM1950 cryostat to ensure comprehensive coverage of all potential output regions. Viral transfection distribution was evaluated by systematically analyzing every fifth consecutive brain slice.

### Fluorescence imaging

The brain tissue sections were subjected to three consecutive washes with PBS and subsequently mounted using an anti-fade mounting medium containing 4',6-diamidino-2-phenylindole (DAPI; P0131, Beyotime). Viral transduction efficiency was assessed through the detection of cherry fluorescence signals using fluorescence microscopy, with co-labeling of the calcium/calmodulin-dependent protein kinase II alpha (CaMKIIα) marker serving as a validation parameter. For immunostaining procedures, anterior cingulate cortex (ACC) sections exhibiting viral expression were permeabilized with 0.5% Triton X-100 in PBS at ambient temperature for 60 min. The sections were then incubated with primary antibodies (rabbit anti-CaMKIIα, 1:250 dilution, 13730–1-AP, Proteintech) at 4 °C for 24 h. Following three PBS washes, the samples were treated with fluorescent secondary antibodies (goat anti-rabbit IgG, 1:650 dilution, 111–545-003, Jackson ImmunoResearch Laboratories) at room temperature for 2 h. After final PBS washes, the sections were mounted with anti-fade medium. Fluorescence imaging was performed using three distinct channels: the 555 nm laser line for visualization of cherry fluorescence, indicating viral-transfected neuronal somata and axonal projections; the 360 nm laser line for DAPI staining, providing anatomical context; and the 488 nm laser line for CaMKIIα immunoreactivity, confirming the neuronal identity of transduced cells.

For fluorescence imaging, comprehensive overview images of the brain were acquired using a 10 × objective lens on a fluorescence scanner (KF-FL-005, China). To achieve higher resolution and detailed visualization, high-magnification images were obtained using a Leica DMi8 inverted fluorescence microscope. Image post-processing was conducted using Leica LAS X software, followed by the application of Adobe Illustrator to accurately delineate brain regions in accordance with the reference brain atlas.

## Results

### AAV virus can effectively and specifically transfer to glutamatergic neurons

**Fig. 1:**
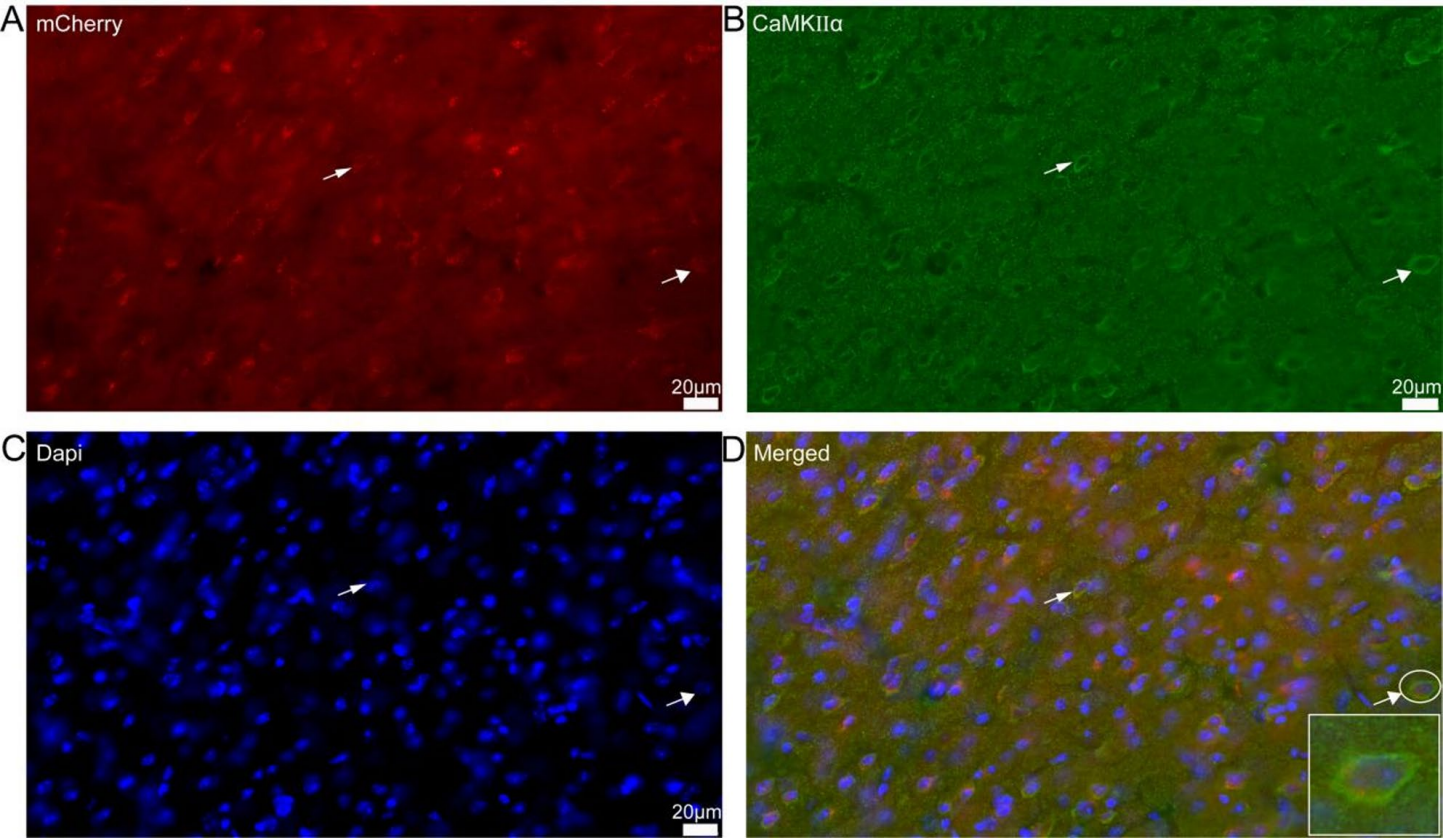
AAV virus effectively and selectively labels glutamatergic neurons. **A** red indicated AAV virus-labeled neurons. **B** green marked the glutamatergic neurons. **C** nuclear staining. **D** co-labeling of virus-transfected and glutamatergic neurons

The transfection efficiency of AAV-CaMKIIα-mCherry-WPREs was assessed by immunofluorescence co-labeling with an antibody against CaMKIIα, a specific marker for excitatory glutamatergic neurons in the cortex. The viral construct demonstrated specific labeling of glutamatergic neurons, as evidenced by distinct fluorescent signals observed under fluorescence microscopy, thereby confirming precise targeting and integration within the neuronal population. The CaMKIIα marker, visualized as bright green fluorescence (Fig. 1B), exhibited substantial colocalization with the red fluorescence emitted by the AAV-expressed reporter protein (Fig. 1A), validating the specificity of the viral transfection for glutamatergic neurons (Fig. 1D). This dual-labeling approach provided robust visual confirmation of the AAV's ability to selectively infect and express genetic material within the targeted neuronal subtype, ensuring the reliability of subsequent experimental interventions.

### Overview of whole-brain efferent projections from glutamatergic neurons in the bilateral anterior cingulate cortex (ACC)

**Fig. 2:**
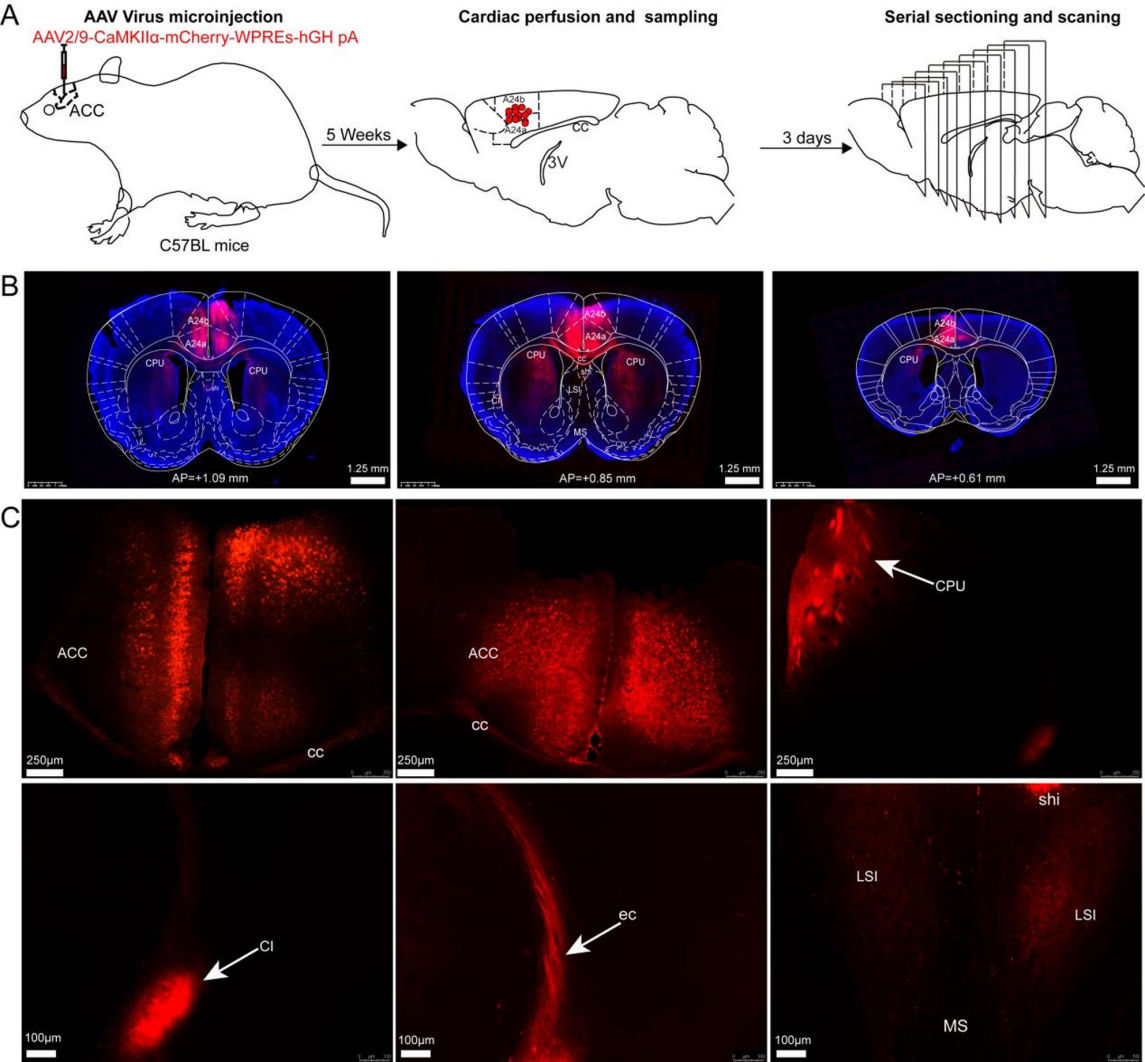
Viral expression and anterograde fiber labeling in bilateral ACC glutamatergic neurons. **A** Schematic of anterograde tracing methodology was used to map projections from bilateral A24a and A24b subregions. **B** fluorescence imaging of whole-brain sections highlighting the ACC region. **C** viral expression was detected in the ACC, with labeled fibers observed in the CPu, cc, ec, CI, MS, LSI, and SHi

The AAV-CaMKIIα-mCherry-WPREs viral vector was employed as a direct projection tracer for targeted neuronal populations. To specifically labelled glutamatergic neurons in the ACC, bilateral stereotaxic injections of the anterograde AAV were administered into subregions A24a and A24b of four adult male C57BL/6 J mice, enabling comprehensive mapping of their projection fibers throughout the brain. The experimental protocol was schematically illustrated in Fig. 2A. Following a five-week incubation period to ensure complete viral expression and axonal transport, two brains were sectioned coronally and two brains were sectioned sagittally to facilitate analysis of both rostro-caudal distributions and mediolateral connectivity patterns. The AAV virus was successfully expressed in the soma of glutamatergic neurons in the bilateral ACC brain region (Fig. 2C). Anatomical localization was performed using the fourth edition of the mouse brain atlas as a reference framework. Figure 2C revealed dense projection fibers from ACC glutamatergic neurons to multiple brain regions, including the corpus callosum (cc), caudate putamen (CPU), external capsule (ec), claustrum (CI), lateral septal nucleus (LSI), medial septal nucleus (MS), and septohippocampal nucleus (SHi).

**Fig. 3:**
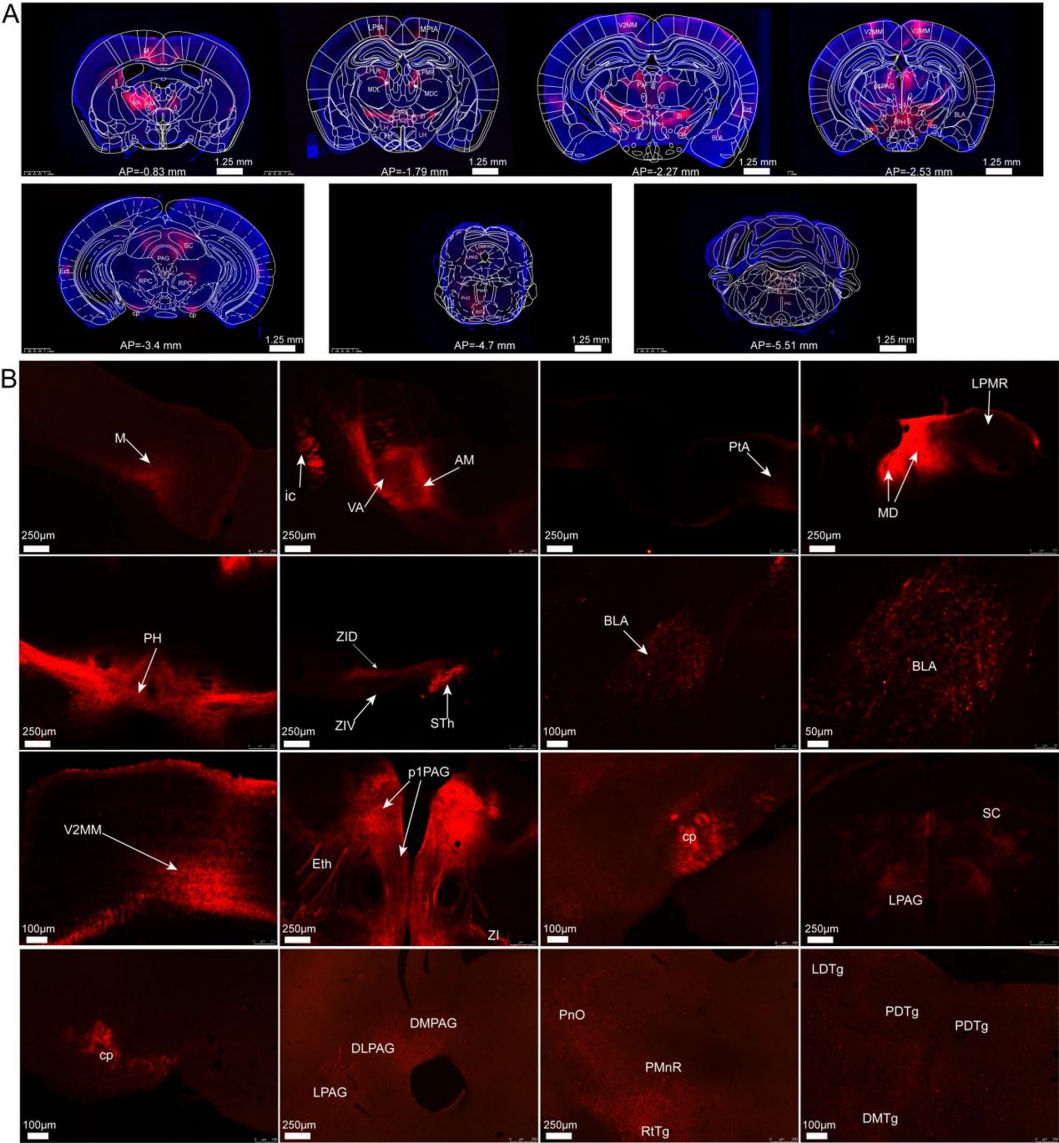
Spatial distribution of glutamatergic neuron projections in bilateral ACC. **A** the coronal plane revealed the anterior–posterior projection pattern of ACC glutamatergic neurons, demonstrating extensive innervation of cortical regions, thalamic nuclei, hypothalamic areas, brainstem structures, and limbic regions. **B** high-magnification imaging highlighted the intricate axonal networks formed by ACC glutamatergic projections. Viral tracing identified labeled fibers in specific subregions, including cortical areas (M, MPtA, LPtA, V2MM, Ect), thalamic nuclei (AM, VA, MDC, MDL, LPLR, LPMR, STh, Re, PaF, ZI), hypothalamic regions (PH, LH), limbic structures (basolateral amygdaloid nucleus), midbrain areas (PAG, SC), and brainstem nuclei (RPC, PnO, PMnR, RtTg, DMTg, LDTg, PDTg)

In the anterior–posterior orientation, Fig. 3A demonstrated the elaborate axonal network emanating from glutamatergic neurons in the ACC, exhibiting a sophisticated and precisely organized projection architecture. Figure 3B showed ACC glutamatergic neurons projected to multiple brain regions: cortical areas, thalamus, hypothalamus, brainstem, subcortical telencephalon (anterior basolateral amygdaloid nucleus, septal nucleus). Notably, we identified cortical inputs derived from the ACC, with direct projections from ACC glutamatergic neurons extending to the motor cortex (M), secondary mediomedial visual cortex (V2MM), medial parietal association cortex (MPtA), lateral parietal association cortex (LPtA), and ectorhinal cortex (Ect).

Glutamatergic neurons originating from the ACC exhibited predominant innervation patterns targeting the thalamic and hypothalamic regions, with particularly dense axonal projections observed in the internal capsule, which anatomically enveloped the thalamic structure. Within the anterior thalamus, ACC efferent fibers were specifically identified in the anteromedial thalamic nucleus (AM) and ventral anterior thalamic nucleus (VA). These projections extended to multiple ventral thalamic nuclei, including the mediodorsal thalamic nucleus (central part, MDC; lateral part, MDL), lateral posterior thalamic nucleus (laterorostral, LPLR; mediorostral, LPMR), subthalamic nucleus (STh), and reuniens thalamic nucleus (Re). In more posterior sections, the parafascicular thalamic nucleus (PaF) was observed to receive substantial ACC fiber projections. Additionally, the zona incerta (ZI), a ventral thalamic region characterized by both inhibitory GABAergic and excitatory glutamatergic neuronal populations, was found to receive significant glutamatergic input from the ACC.

In hypothalamic brain slice preparations, neural fibers were distinctly localized within the posterior hypothalamic nucleus (PH) and lateral hypothalamus (LH). The prosomere1 periaqueductal gray (p1PAG) was anatomically identified in the rostral midbrain, positioned anterior to the caudal PAG structures within the tegmental region surrounding the cerebral aqueduct. Neuroanatomical tracing revealed that glutamatergic neurons projected to the p1PAG, PAG, and superior colliculus (SC). Additionally, virual infected fibers were observed in multiple brainstem nuclei, including the oral pontine reticular nucleus (PnO), paramedian raphe nucleus (PMnR), reticulotegmental nucleus (RtTg), dorsomedial tegmental area (DMTg), laterodorsal tegmental nucleus (LDTg), and posterodorsal tegmental nucleus (PDTg).

### Overview of whole-brain efferent projections from glutamatergic neurons in the bilateral midcingulate cortex (MCC)

The AAV vector, engineered to express a cherry fluorescent protein tag for visualization, was bilaterally microinjected into the precise anatomical coordinates corresponding to the A24a' and A24b' subregions of the MCC, ensuring accurate targeting of the glutamatergic neuron population in these areas. To investigate whole-brain projections of MCC glutamatergic neurons, we administered an AAV vector into the bilateral MCC of four adult male mice.

**Fig. 4:**
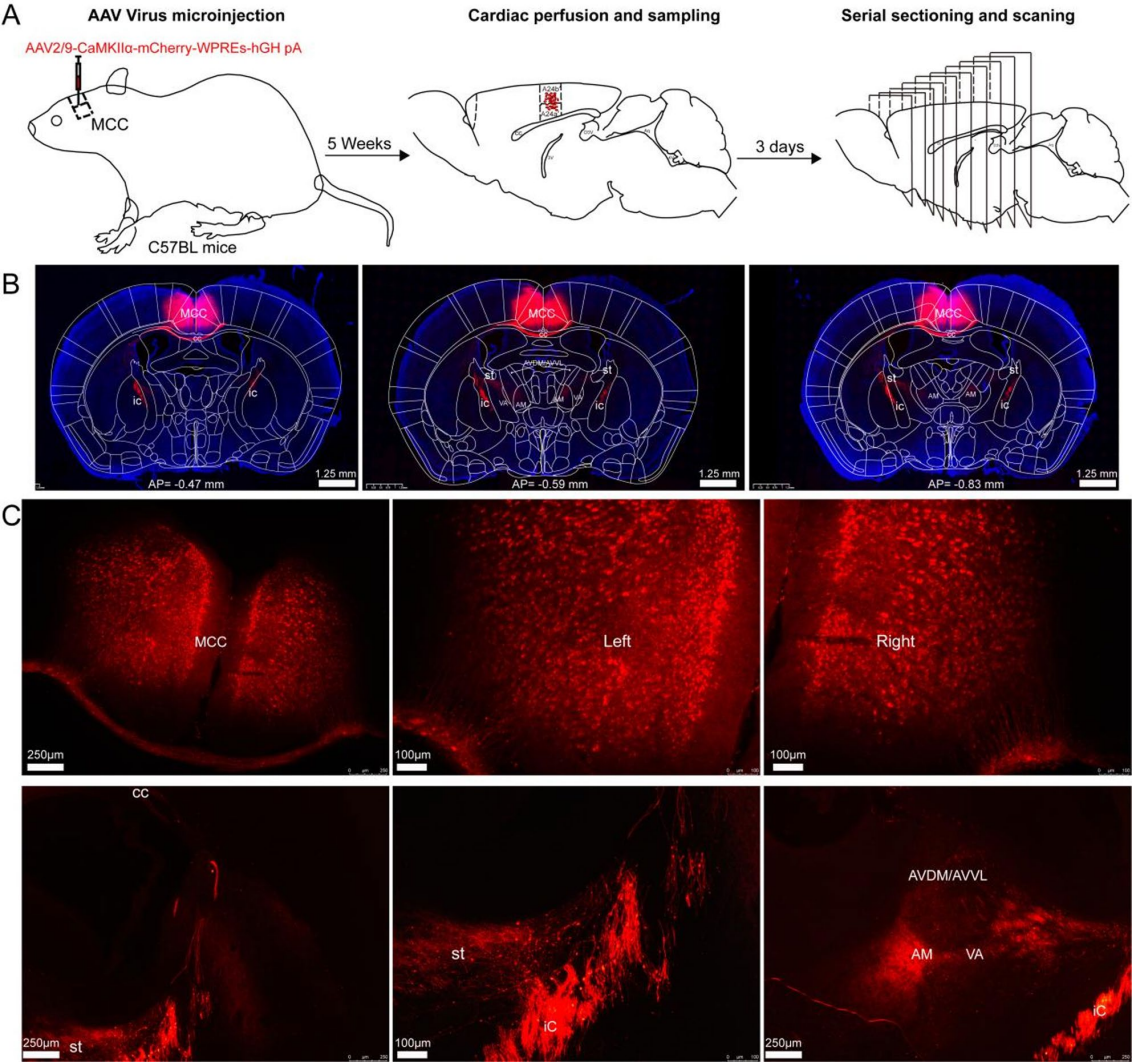
Viral expression and anterograde fiber labeling in bilateral MCC glutamatergic neurons. **A** the procedure for anterograde tracing projections from the bilateral A24a'/A24b'. **B** Whole-brain fluorescence images of brain slices encompassing the MCC region. **C** viral expression in MCC glutamatergic neurons was observed, along with the distribution of virus-infected fibers in the cc, st, ic, and thalamus

**Fig. 5:**
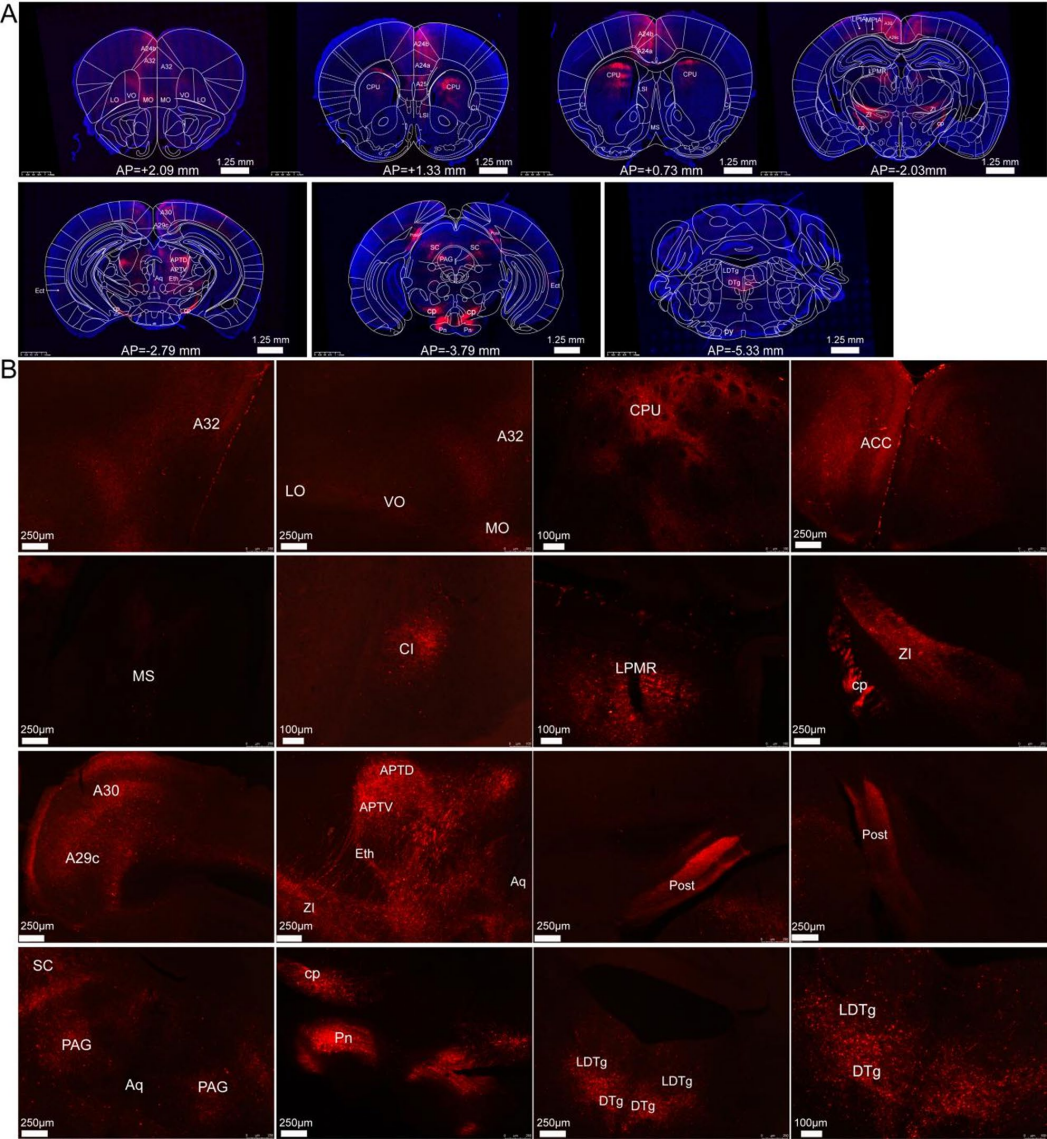
Spatial distribution of glutamatergic neuron projections in bilateral MCC. **A** axonal projection patterns of MCC glutamatergic neurons across anterior–posterior coronal sections. **B** virus-infected fibers were distributed in the A32, LO, VO, MO, ACC (A24a and A24b), CPU, CI, LPMR, ZI, CP, PCC (A29c and A30), APTD, APTV, Eth, Post, SC, PAG, Pn, LDTg, and DTg

The experimental protocol is schematically depicted in Fig. 4A. Whole-brain sections encompassing the MCC are presented in Fig. 4B. AAV vector expression was observed in glutamatergic neuron somata of the bilateral MCC (Fig. 4C). Robust anterograde fluorescent labeling revealed dense projections concentrated within thalamic regions, demonstrating continuous axonal trajectories traversing the corpus callosum (cc), internal capsule (ic), and stria terminalis (st). The st is a critical white matter tract that serves as a principal conduit for neural information transmission. Our analysis identified that the thalamic nuclei, including the anteromedial (AM), ventral anterior (VA), anteroventral dorsomedial (AVDM), and anteroventral ventrolateral (AVVL) nuclei, received inputs from the MCC via the st (Fig. 4C). Additionally, virus infected fibers were observed terminating in the lateral posterior thalamic nucleus, mediorostral part (LPMR) and zona incerta (ZI) (Fig. 5B).

Anterograde tracing revealed extensive projections of glutamatergic neurons from the MCC across multiple cortical regions (Fig. 5), including the medial orbital cortex (MO), ventral orbital cortex (VO), lateral olfactory tract (LO), anterior cingulate cortex (subdivisions A24a and A24b), and posterior cingulate cortex (areas 29 and A30). Projections were also observed in basal forebrain structures, specifically the medial septal nucleus (MS) and the claustrum (CI). Viral-labeled fibers were densely localized around the cerebral aqueduct, encompassing the periaqueductal gray (PAG), anterior pretectal nucleus (APT), and superior colliculus (SC). In the brainstem, labeled fibers were identified in the pontine nuclei (Pn), laterodorsal tegmental nucleus (LDTg), and dorsal tegmental nucleus (DTg). Furthermore, viral-infected fibers were detected in the postsubiculum (Post).

### Overview of whole-brain efferent projections from glutamatergic neurons in the bilateral posterior cingulate cortex (PCC)

**Fig. 6:**
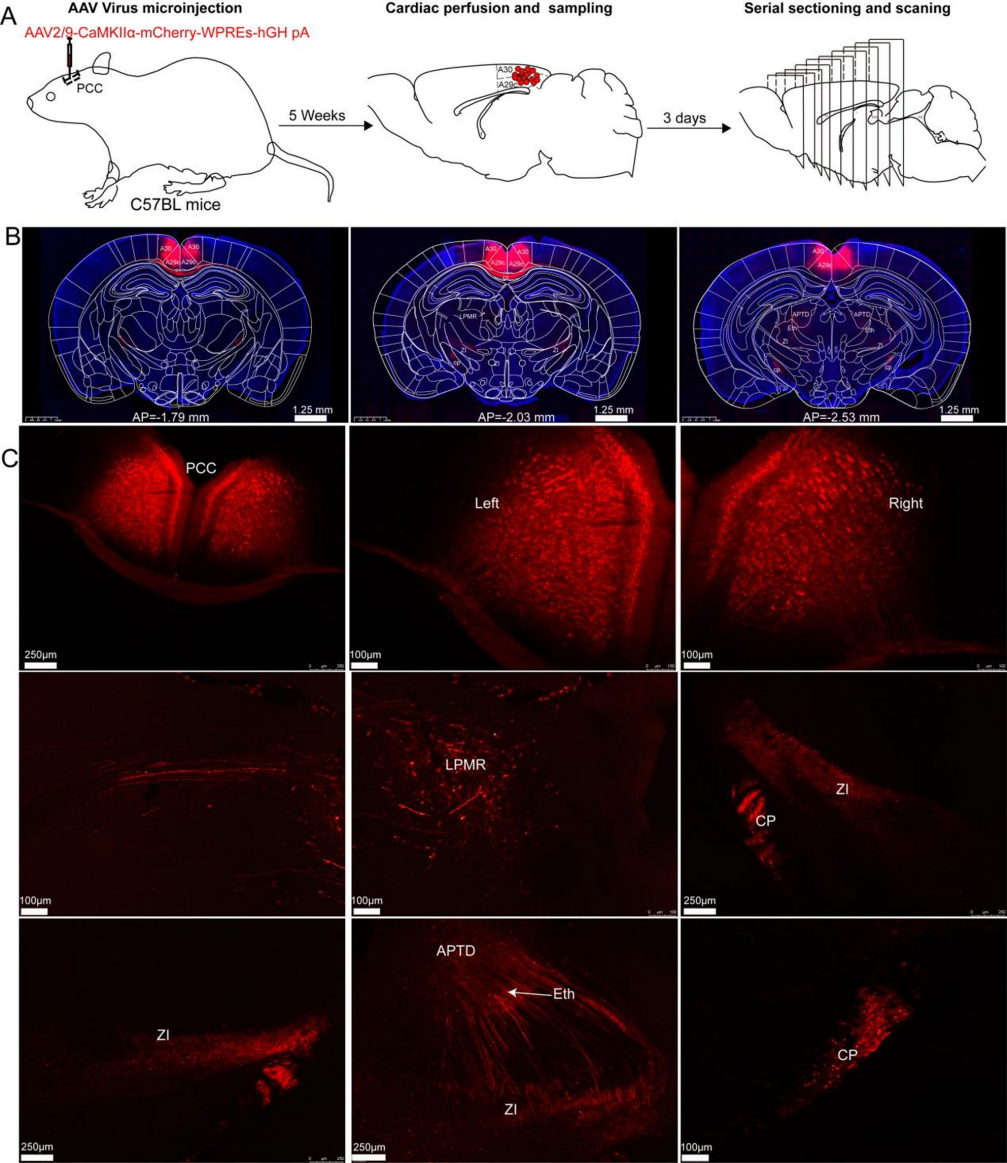
Viral expression and anterograde fiber labeling in bilateral PCC glutamatergic neurons. **A** the procedure for anterograde tracing projections from the bilateral A30 and A29c. **B** Whole-brain fluorescence images of brain slices encompassing the PCC region. **C** viral was expressed in PCC glutamatergic neurons and the distribution of virus-infected fibers was observed in the cc, LPMR, ZI, cp, Eth, and APTD

To examine the efferent projections of glutamatergic neurons in the PCC, we performed stereotaxic injections of AAV virus into the bilateral PCC, with precise targeting of subregions A29c and A30. Figure 6A schematically showed the experimental design. Figure 6B illustrated the distribution of projection fibers throughout whole-brain sections, encompassing the PCC. Successful viral expression was confirmed in the somata of PCC glutamatergic neurons (Fig. 6C), with dense labeled fibers observed in the zona incerta (ZI) and cerebral peduncle (cp). Additionally, virus-labeled fibers were identified in the ethmoid thalamic nucleus (Eth), the mediorostral division of the lateral posterior thalamic nucleus (LRMR), and the dorsal aspect of the anterior pretectal nucleus (APTD).

**Fig. 7:**
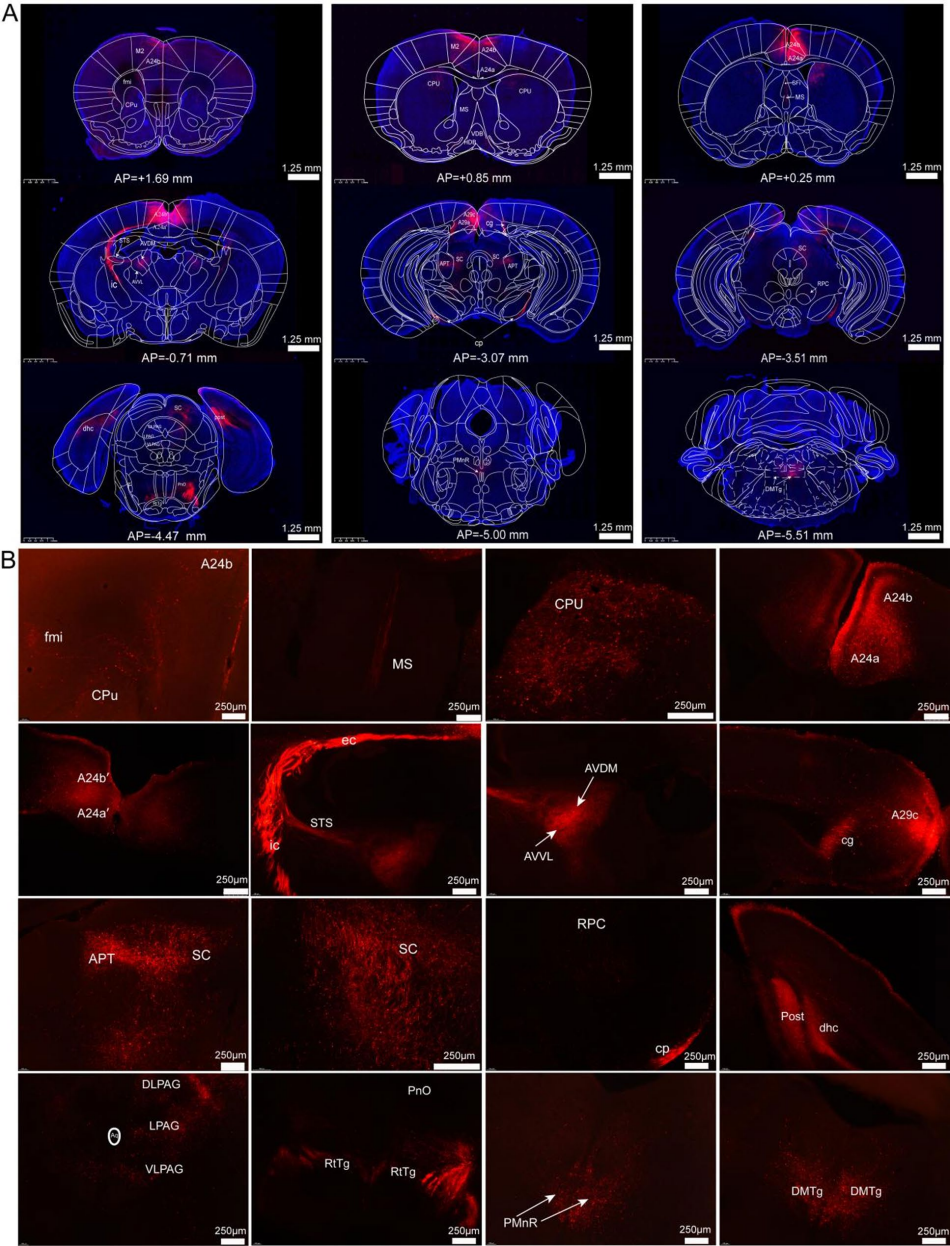
Spatial distribution of glutamatergic neuron projections in bilateral PCC. **A** illustrated the axonal projection patterns of PCC glutamatergic neurons across anterior–posterior coronal sections. **B** the distribution of virus-infected fibers in the ACC (including A24a and A24b subregions), the MCC (including A24a' and A24b' subregions), CPu, and septal nucleus, thalamic regions, brainstem nucleus as well as limbic regions (post and dhc)

The PCC glutamatergic neurons exhibited extensive connectivity with multiple cerebral regions, including various cortical areas, thalamic nuclei, subcortical telencephalic structures, brainstem nuclei, and limbic regions, as illustrated in Fig. 7. Virus infected fibers were found in cortical areas, notably the secondary motor cortex (M2), primary and secondary visual cortices (V1 and V2), anterior cingulate cortex (A24a/A24b), and middle cingulate cortex (A24a'/A24b'). Furthermore, PCC glutamatergic neurons established direct projections via the stria terminalis (st) to the anteroventral thalamic nucleus, specifically targeting its ventrolateral (AVVL) and dorsomedial (AVDM) subdivisions. Anterograde tracing revealed dense AAV-labeled fibers in the caudate putamen (CPu) traversing corpus callosum (cc), as well as axonal labeling in the medial septal nucleus (MS), nucleus of the vertical limb of the diagonal band (VDB), nucleus of the horizontal limb of the diagonal band (HDB), and septofimbrial nucleus (SFi). Robust PCC projections were observed in periaqueductal regions, including the superior colliculus (SC), anterior pretectal nucleus (APT), and periaqueductal gray (PAG). Additionally, the cerebral peduncle (cp) received substantial PCC inputs, which were subsequently relayed to the middle cerebellar peduncle (mcp), reticulotegmental nucleus of the pons (RtTg), dorsomedial tegmental area (DMTg), paramedian raphe nucleus (PMnR), and parvicellular part of the red nucleus (RPC) in the brainstem. Within the limbic system, dense labeling was identified in the postsubiculum (Post) and dorsal hippocampal commissure (dhc).

## Discussion

**Fig. 8:**
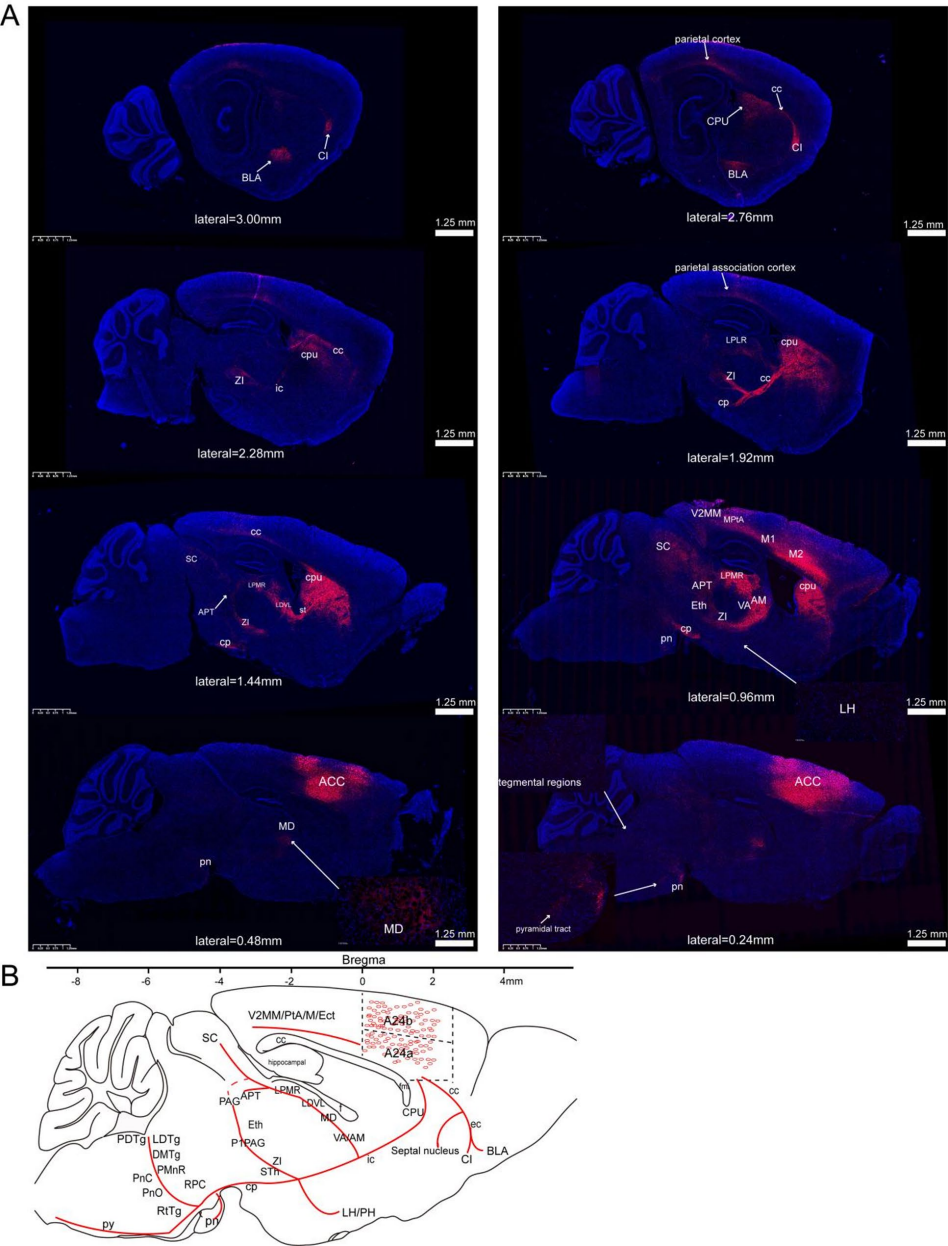
Comprehensive mapping of whole-brain efferent projections from bilateral ACC glutamatergic neurons. **A** axonal projection patterns of ACC glutamatergic neurons were visualized through sagittal whole-brain section imaging. **B** systematic reconstruction of whole-brain efferent connectivity was performed to delineate the projection patterns of ACC glutamatergic neurons

**Fig. 9:**
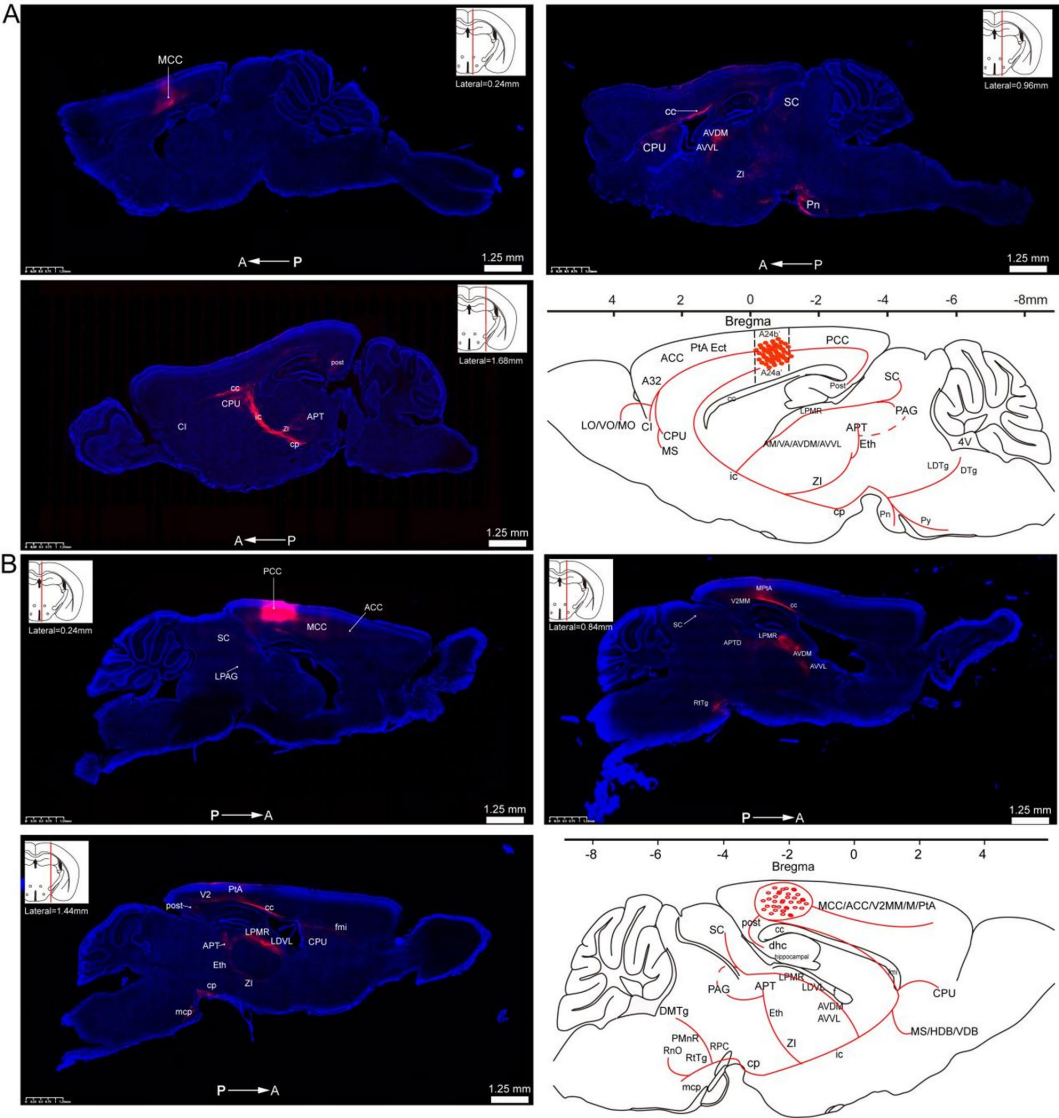
A comprehensive overview of whole-brain efferent projections from glutamatergic neurons in the bilateral MCC and PCC. **A** illustrated sagittal fluorescence imaging, depicting the whole-brain outputs and the reconstructed projection patterns of MCC glutamatergic neurons. **B** provided sagittal views detailing whole-brain axonal distributions and reconstructed projection patterns of PCC glutamatergic neurons

The cingulate cortex was systematically classified into three distinct regions along the rostrocaudal axis in mammals through homologous nomenclature: the anterior cingulate cortex (ACC, A24a/A24b), midcingulate cortex (MCC, A24a'/A24b'), and posterior cingulate cortex (PCC, A30/A29c) [16]. This classification framework has facilitated the anatomical and functional characterization of the MCC as an independent structural entity. In this study, we utilized homologous nomenclature to investigate the whole-brain projections of glutamatergic neurons within the cingulate cortex. An anterograde viral tracer was microinjected into the bilateral ACC, MCC, and PCC of adult mice, and fluorescence imaging was performed on a series of coronal and sagittal sections to reconstruct the global projection patterns. Viral tracing and fluorescence imaging demonstrated that glutamatergic neurons in the bilateral ACC (Fig. 8), MCC (Fig. 9), and PCC (Fig. 9) exhibited a conserved, widespread projection pattern mediated by three principal axonal pathways. The corpus callosum (cc) enabled interhemispheric communication. Descending projection systems of the internal capsule (ic) and cerebral peduncle (cp) innervated subcortical and brainstem structures.

The cc represents the most extensive white matter structure in the brain, comprising densely organized nerve fiber bundles. Utilizing brain slice scanning and fluorescence tracing techniques, we identified that cingulate glutamatergic neurons extend axonal projections through the cc to multiple regions, including the caudate putamen (CPU), claustrum (CI), orbitofrontal cortex, and other associated areas. The ic, another critical white matter structure, consists of tightly packed nerve fiber bundles that facilitate neural connectivity. Cingulate glutamatergic neurons established subcortical connections via the ic, which exhibited dorsal projections to the thalamus, anterior pretectal nucleus (APT), superior colliculus (SC), and periaqueductal gray (PAG), as well as ventral projections to the zona incerta (ZI), hypothalamus, and cp. Notably, the cp served as a direct caudal extension of the ic, projecting to brainstem nuclei such as the dorsal medial tegmental area (DMTg), retrotrapezoid nucleus (RtTg), laterodorsal tegmental nucleus (LDTg), and pontine nuclei (Pn). Observations showed significant differences in downstream projections of ACC, MCC, and PCC, aligning with their functional specializations. This suggests distinct output connectivity patterns underlie their specialized roles.

We further compared the outputs of the bilateral ACC/MCC/PCC glutamatergic neurons with existing literature and investigated their functional contributions. This study provided a comprehensive analysis of the whole-brain efferent projections of cingulate glutamatergic neurons, emphasizing their neural connectivity and functional significance.

### Cerebral cortex

Extensive anatomical studies in monkeys, rabbits, and rats showed consistent neural connections between the ACC and multiple cortical regions [17–19]. Specifically, ACC neurons have been shown to establish efferent projections to diverse cortical areas, encompassing the motor, visual, orbitofrontal, and somatosensory cortices, as well as the limbic regions. Based on this evidence, we systematically mapped the projection patterns of glutamatergic neurons originating from the ACC, MCC, and PCC to their respective cortical target regions.

Our findings demonstrated that the bilateral ACC glutamatergic neurons projected to multiple cortical regions, including the motor cortex (M), secondary mediom visual cortex (V2MM), medial parietal association cortex (MPtA), lateral parietal association cortex (LPtA), and ectorhinal cortex (Ect). These observations aligned with a previous study using the virus anterograde tracing technique and an ultrahigh-speed imaging method [20]. The identified anatomical connections between the ACC and both motor and visual processing areas suggest a potential role for the ACC in decision-making processes, particularly in the integration of visual information [21, 22]. Furthermore, existing literature has comprehensively reviewed the neuronal representation of spatial information within the parietal association cortex (PtA) [23]. The glutamatergic projections originating from the ACC to the PtA were hypothesized to play a critical role in the integration of spatial information, including location, distance, and directional cues within the environment [24]. In contrast, the MCC exhibited a distinct projection pattern, as it did not innervate the motor, or visual cortex. However, virus-infected fibers from MCC were identified in the orbitofrontal cortex (LO, VO, and PO), ACC, PCC, and postsubiculum (post), indicating that MCC glutamatergic neurons modulated motivational behavior [25, 26], spatial navigation, and memory [27].

Our study revealed distinct characteristics of intra-cingulate connectivity in mice. The cingulate cortex was found to possess a complex network architecture, with the PCC demonstrating particularly robust glutamatergic projections that establish reciprocal connections with both the ACC and MCC. Notably, specific subregions, particularly A24a/A24b and A24a'/A24b', exhibited pronounced cherry fluorescence signals, characterized by high signal intensity and well-defined interregional connectivity patterns. Besides, PCC also output to M2, post and dorsal hippocampal commissure (dhc). Monosynaptic rabies virus tracing demonstrates that excitatory neurons in the dorsal subiculum receive robust intrinsic inputs from the retrosplenial cortex (areas A30/A29) [28]. Furthermore, our findings indicate that PCC glutamatergic neurons can project to post-subiculum (post). The post, a vital structure situated between the hippocampus and the entorhinal cortex, significantly contributed to spatial navigation, movement, memory processing, and the regulation of the stress response [29]. These observations suggest that the PCC-post neural circuit may represent a fundamental substrate underlying the pathophysiology of learning and memory impairments.

### Non-cortical telencephalon

The non-cortical telencephalon primarily includes the basal forebrain, basal ganglia, amygdala, which play a crucial role in motor control, autonomic functions, attention, memory, cognitive and emotion, forming a complex interactive network with the cortex of the brain [30–33]. In the current investigation, glutamatergic neuronal fibers originating from the bilateral ACC glutamatergic neurons projected to the basolateral amygdaloid nucleus (BLA), dorsal CPU, CI, and septal regions. The existence of ACC neuronal projections to the BLA remains a subject of scientific debate. While previous studies have documented ACC-BLA projections in both mice and monkeys [13, 34], Shi et al. reported an absence of such projections to the BLA or hippocampus in mice [20]. This discrepancy may be attributed to methodological differences, particularly the duration of anterograde viral expression. Shi et al. collected samples at three weeks post-injection, whereas our study used a five-week post-injection time point. Our neuroanatomical analysis using sagittal and coronal brain sections showed consistent projections of glutamatergic neurons from ACC to BLA. Furthermore, the functional significance of the ACC-BLA circuit has been established, with Yuan et al. demonstrating its involvement in modulating reward intake, devaluation processes, and depression-like behaviors [35]. Specifically, inhibition of the cortico-amygdalar pathway was shown to reduce sensitivity to reward devaluation while increasing reward consumption. Notably, our findings showed no virus-labeled fiber projections from glutamatergic neurons in the MCC and PCC to the BLA.

The CPU is a primary striatal target for ACC projections, consistent with a prior mouse study on cingulate-striatum connectivity [36]. We further observed robust projections from both the MCC and PCC terminating in the CPU. These anatomical findings establish the CPU as a major convergence zone for cingulate cortex outputs. Notably, functional studies have implicated cingulate-striatal circuits in specific behaviors. For example, Li et al. [37] demonstrated that ACC pyramidal neurons projecting to the dorsomedial striatum (a subregion of the CPU) play a pivotal role in modulating pain-associated insomnia. In their study, chemogenetic inhibition of this specific pathway ameliorated chronic pain-induced insomnia in mice.

The CI is a thin gray matter layer in the telencephalon, located between the putamen and insular cortex [38]. Our anatomical investigations shown that the CI received dense projections from the ACC and MCC via the external capsule, but not from the PCC. Accumulating evidence has demonstrated that the CI represented a densely interconnected neural architecture, functioning as a pivotal hub for consciousness, attention, and the integration of cross-modal sensory information [39, 40]. Notably, Luo et al. [41] revealed that CI neurons, particularly through their GABA_A_ receptor-mediated mechanisms, played a pivotal role in propofol-induced unconsciousness, as substantiated by comprehensive behavioral assessments and electroencephalographic (EEG) analyses. These collective findings suggest that the cingulate-claustral projection pathway may be involved in the modulation of transitions between conscious states.

Finally, researches on anatomical functional connectivity indicated the medial septal nucleus (MS)-hippocampal circuit modulated spatial memory deficits and may be a potential treatment for cognitive impairments [42, 43]. In our study, the MS was targeted by the ACC, MCC, and PCC. In addition to the MS, light cherry fibers from ACC were observed in lateral septal nucleus (LSI) and septohippocampal nucleus (SHi). However, the precise functional mechanisms underlying the cingulate-septal nucleus circuit are not fully understood and require further investigation to be elucidated.

### Thalamus

The cingulothalamic projections constituted essential components of neural circuitry, facilitating bidirectional communication between the cingulate cortex and the thalamus. Although anterograde and retrograde viral tracing techniques have been used to study thalamic-anterior cingulate projections [15, 44], the specific projections of glutamatergic neurons in bilateral cingulate subregions remain insufficiently characterized and require further investigation.

This study systematically examined the efferent projections of glutamatergic neurons from the ACC, MCC, and PCC to thalamic nuclei. ACC glutamatergic neurons were observed to extend axonal projections through the corpus callosum and internal capsule, terminating in specific thalamic nuclei, including the anteromedial (AM), ventral anterior (VA), lateral posterior (LP), and mediodorsal (MD) nuclei. Viral tracing revealed that MCC-derived fibers innervated the VA and AM nuclei, whereas the PCC exhibited no such projections. Notably, the anteroventral ventrolateral (AVVL) and anteroventral dorsomedial (AVDM) nuclei received inputs from the MCC and PCC but not from the ACC. These findings revealed distinct cortico-thalamic projection patterns across cingulate subregions, offering critical anatomical insights into this circuitry. While the functional modulation of cingulate-thalamic pathways remains incompletely elucidated, neurophysiological and clinical evidence underscores their involvement in diverse processes, including pain, emotion, and itch sensation. For example, deep brain stimulation of thalamic nuclei has been shown to modulate ACC neuronal activity, suggesting a potential mechanism for alleviating chronic neuropathic pain [3, 45].

The MD-to-ACC pathway was implicated in regulating observational fear-related activity [46], while the AM nucleus acted as a critical "itch gate," relaying itch-specific unpleasantness to the cingulate cortex [47]. Furthermore, MD exerted a desynchronizing effect on seizure-like activity in the cingulate cortex, primarily by modulating the firing patterns of pyramidal neurons [48].

Our findings further demonstrated that glutamatergic neurons in the cingulate cortex exhibited dense projections to the ZI, a subthalamic structure. The ZI, characterized as a small, plate-like nucleus situated in the ventral thalamic region, has been increasingly implicated in diverse neurobehavioral processes. Recent evidence highlighted its pivotal role in fear conditioning, memory consolidation, and the modulation of innate defensive responses [49]. Additionally, the ZI has been shown to influence early social-emotional development [50] and regulate circadian sleep–wake transitions [51]. These observations collectively suggest that the ZI serves as a critical neural hub for mediating top-down regulatory processes originating from the cingulate cortex. A comprehensive investigation is warranted to elucidate the precise functional mechanisms underlying the cingulate-ZI neural circuitry.

### Hypothalamus

A previous study identified a direct neural projection from the dorsal ACC to the lateral hypothalamus (LH) in rodent models [52]. Our investigation revealed that the posterior hypothalamic area (PH) is the target of bilateral ACC glutamatergic projections, with only minimal fiber innervation in the LH. Notably, after optimizing fluorescence intensity and contrast parameters, we found no evidence of projections from the MCC or PCC to hypothalamic regions. Emerging evidence positions the hypothalamus as a critical node in neural networks governing multiple physiological processes, including neuropathic pain modulation [53], reward processing related to drugs and food [54], and the regulation of arousal states and consciousness [55]. These findings collectively contribute to our understanding of the functional significance of the ACC-hypothalamus circuit in the pathophysiology of various disorders, particularly neuropathic pain, substance abuse, and hypersomnia.

### Brainstem

Viral tracing data confirmed prior findings of the cingulate cortex efferent projections to the brainstem. Sagittal plane analysis revealed the ventral internal capsule branch extends through the cerebral peduncle to the brainstem.

A monosynaptic retrograde tracing study has demonstrated that ACC neurons established direct innervation with cholinergic neurons in the laterodorsal tegmental nucleus (LDTg) [56]. Furthermore, our investigation showed the bilateral ACC and MCC glutamatergic neurons exhibited direct projections to the LDTg. A research has established that sensorimotor cortices projected to pontine nuclei (Pn) and red nuclei, which served as critical hubs mediating connectivity between the cerebral cortex and cerebellum, playing essential roles in motor regulation [57]. Our findings additionally demonstrated that the ACC projected to the oral pontine nucleus (PnO, PnC, and Pn), while the MCC only projected to the Pn. Moreover, glutamatergic neurons in the ACC and PCC, but not MCC sent projections to the parvicellular part of the red nucleus (RPC). The paramedian raphe nucleus (PMnR) has been identified as a critical regulatory hub for arousal and motor brainstem nuclei, as evidenced by a human fMRI study [58]. Activation of CaMKIIα-expressing neurons in the PMnR induced a rapid transition from wakefulness to general anesthesia, whereas inhibition of these neurons significantly reduced the loss of righting reflex (LORR) rate in mice [59]. These findings collectively suggest that the PMnR plays a pivotal role in modulating arousal suppression and motor function. Our findings demonstrated that the PMnR received glutamatergic inputs from the ACC and PCC, with no detectable projections originating from the MCC. While these anatomical connections have been established, their functional significance remains to be elucidated through further investigation. Furthermore, Chen et al. [60] has provided substantial evidence that ACC stimulation directly facilitated spinal sensory excitatory transmission. However, our study did not characterize specific projections from cingulate cortex glutamatergic neurons to the spinal cord, which is a significant future research avenue.

Neuroanatomical evidence collectively shows that cingulate-brainstem circuits position the cingulate cortex as a hierarchical modulator of multiple brainstem nuclei. Specifically, the cingulate cortex exerts top-down regulatory control over the SC, influencing instinctive behaviors and cognitive processes [61]; the PAG, modulating innate defensive responses and neuropathic pain processing [62, 63]; and the tegmental nucleus, regulating addiction-related behaviors, sleep–wake cycles, and respiratory functions [64, 65].

## Conclusion

In this study, we utilized adeno-associated virus-based tracing to generate the first comprehensive map of glutamatergic projections originating from the bilateral ACC (areas 24a/24b), MCC (areas 24aʹ/24bʹ), and PCC (areas A30/A29c) in mice. Although these regions are anatomically distinct, the ACC, MCC, and PCC glutamatergic neurons exhibit a conserved projection signature, characterized by efferent connections to other cingulate subregions, widespread cortical areas, and key telencephalic structures, as well as to the thalamus, and brainstem. This shared connectivity profile supports the integrated processing of pain perception, emotional regulation, memory consolidation, and autonomic control. Functional segregation is evident through the distinct engagement of large-scale networks via specialized subcortical and cortical output pathways, underscoring the modular organization of neural circuits. The ACC glutamatergic neurons may regulate affective and instinctive states through specialized neural circuits involving the BLA, CI, LH, and PH, which are critically implicated in emotional processing, conscious state modulation, and addiction-related behaviors. The MCC glutamatergic neurons may serve to integrate motivational signals with action planning and spatial processing, via its projections to the orbitofrontal cortex, anteroventral thalamic nucleus, and post-subiculum. The PCC glutamatergic neurons are connected to the post-subiculum, red nucleus, and hippocampal commissure, thereby supporting spatially oriented motor control and contextual memory encoding.

Our study has several technical limitations. First, our analysis was primarily qualitative. We focused on describing the spatial distribution patterns of anterogradely labeled fibers but did not perform a quantitative assessment of fluorescence signal intensity or fiber density. While the use of standardized protocols across all animals enhances reproducibility, the lack of quantitative metrics may limit the precision of comparisons between different projection targets or experimental conditions in future studies. Second, the viral tracing approach employed here does not differentiate between ipsilateral and contralateral projections. To efficiently generate a comprehensive whole-brain projection atlas from the cingulate cortex, we performed bilateral injections. Consequently, the resulting projection maps represent a composite of inputs from both hemispheres, preventing analysis of potential hemispheric asymmetry in connectivity. Third, the resolution of our imaging, while suitable for mapping axonal trajectories and terminal fields, is insufficient for discerning subcellular structures such as synaptic boutons or spines. This limits our ability to infer the strength or synaptic nature of the identified connections at the ultrastructural level. Future studies could address these limitations by employing complementary techniques. Unilateral injections followed by high-resolution imaging and quantitative analysis would enable the dissection of hemispheric-specific pathways. Furthermore, integrating our approach with advanced volumetric imaging methods, such as VISoR [66], could facilitate accurate 3D reconstruction and quantitative morphological analysis of entire neuronal arbors, ultimately improving the spatial resolution and systematic evaluation of neural circuit connectivity.

Collectively, the neuroanatomical architecture delineated in our study provides a structural framework for understanding how cingulate glutamatergic signaling orchestrates distinct physiological processes. This work serves as a springboard for future investigations to precisely dissect the pathways and functional roles of glutamatergic neurons within the ACC, MCC, and PCC.

## Data Availability

No datasets were generated or analysed during the current study.

## Abbreviations

A24a: Cingulate cortex, area 24a
A24a': Cingulate cortex, area 24a'
A24b: Cingulate cortex, area 24b
A24b': Cingulate cortex, area 24b
A29c: Cingulate cortex, area 29c
ACC: Anterior cingulate cortex (A24a and 24b)
AM: Anteromedial thalamic nucleus
AVDM: Anteroventral thalamic nucleus, dorsomedial
AVVL: Anteroventral thalamic nucleus, ventrolateral
Aq: Aqueduct
APTD: Anterior pretectal nucleus, dorsal part
APTV: Anterior pretectal nucleus, ventral part
BLA: Anterior basolateral amygdaloid nucleus
BLP: Post basolateral amygdaloid nucleus
cc: Corpus callosum
cg: Cingulum
CI: Claustrum
CPu: Caudate putamen (striatum)
Cp: Cerebral pedunclec
Dhc: Dorsal hippocampal commissure
DMTg: Dorsomedial tegmental area
Ec: External capsule
Eth: Ethmoid thalamic nucleus
Fmi: Forceps minor of the corpus callosum
HDB: Nucleus of the horizontal limb of the diagonal band
ic: Internal capsule
LH: Lateral hypothalamus
LSI: Lateral septal nucleus
LPtA: Lateral parietal association cortex
LPLR: Lateral posterior thalamic nucleus, laterorostral
LPMR: Lateral posterior thalamic nucleus, mediorostral
LDTg: Laterodorsal tegmental nucleus
Lo: Lateral olfactory tract
M2: Secondary motor cortex
M: Motor cortex
MCC: Middle cingulate cortex (24a' and 24b')
MDC: Mediodorsal thalamus nucleus, central part
MDL: Mediodorsal thalamus nucleus, lateral part
MO: Medial orbital cortex
MPtA: Medial parietal association cortex
MS: Medial septal nucleus
p1PAG: Prosomere 1 PAG
PAG: Periaqueductal gray
PaF: Parafascicular thalamic nucleus
PH: Posterior hypothalamic nucleus
PnO: Oral pontine reticular nucleus
PMnR: Paramedian raphe nucleus
PDTg: Posterodorsal tegmental nucleus
Pn: Pontine nuclei
PCC: Posterior cingulate cortex (A29c and A30)
Post: Postsubiculum
Re: Reuniens thalamic nucleus
RtTg: Reticulotegmental nucleus of the pons
RPC: Red nucleus, parvicellular part
st: The bed nucleus of the stria terminalis
SC: Superior colliculus
STh: Subthalamic nucleus
SHi: Septohippocampal nucleus
ic: Internal capsule
SFi: Septofimbrial nucleus
VA: Ventral anterior thalamic nucleus
VO: Ventral orbital cortex
V2MM: Secondary mediom visual cortex
V1: Primary visual cortex
VDB: Nucleus of the vertical limb of the diagonal band
ZI: Zona incerta
